# Aird: a computation-oriented mass spectrometry data format enables a higher compression ratio and less decoding time

**DOI:** 10.1186/s12859-021-04490-0

**Published:** 2022-01-12

**Authors:** Miaoshan Lu, Shaowei An, Ruimin Wang, Jinyin Wang, Changbin Yu

**Affiliations:** 1 Zhejiang University, Hangzhou, 310009 Zhejiang Province China; 2 School of Engineering, Westlake University, 18 Shilongshan Road, Hangzhou, 310024 Zhejiang Province China; 3 School of Life Sciences, Westlake University, 18 Shilongshan Road, Hangzhou, 310024 Zhejiang Province China; 4 Institute of Advanced Technology, Westlake Institute for Advanced Study, 18 Shilongshan Road, Hangzhou, 310024 Zhejiang Province China; 5 Institute of Biology, Westlake Institute for Advanced Study, 18 Shilongshan Road, Hangzhou, 310024 Zhejiang Province China; 6 Fudan University, Shanghai, China; 7 Shandong First Medical University & Shandong Academy of Medical Sciences, Jinan, 250000 China

**Keywords:** Mass spectrometry, Aird, Compressor, ZDPD, DIA, DDA, Proteomics, Metabolomics

## Abstract

**Background:**

With the precision of the mass spectrometry (MS) going higher, the MS file size increases rapidly. Beyond the widely-used open format mzML, near-lossless or lossless compression algorithms and formats emerged in scenarios with different precision requirements. The data precision is often related to the instrument and subsequent processing algorithms. Unlike storage-oriented formats, which focus more on lossless compression rate, computation-oriented formats concentrate as much on decoding speed as the compression rate.

**Results:**

Here we introduce “Aird”, an opensource and computation-oriented format with controllable precision, flexible indexing strategies, and high compression rate. Aird provides a novel compressor called Zlib-Diff-PforDelta (ZDPD) for m/z data. Compared with Zlib only, m/z data size is about 55% lower in Aird average. With the high-speed decoding and encoding performance of the single instruction multiple data technology used in the ZDPD, Aird merely takes 33% decoding time compared with Zlib. We have downloaded seven datasets from ProteomeXchange and Metabolights. They are from different SCIEX, Thermo, and Agilent instruments. Then we convert the raw data into mzML, mgf, and mz5 file formats by MSConvert and compare them with Aird format. Aird uses JavaScript Object Notation for metadata storage. Aird-SDK is written in Java, and AirdPro is a GUI client for vendor file converting written in C#. They are freely available at https://github.com/CSi-Studio/Aird-SDK and https://github.com/CSi-Studio/AirdPro.

**Conclusions:**

With the innovation of MS acquisition mode, MS data characteristics are also constantly changing. New data features can bring more effective compression methods and new index modes to achieve high search performance. The MS data storage mode will also become professional and customized. ZDPD uses multiple MS digital features, and researchers also can use it in other formats like mzML. Aird is designed to become a computing-oriented data format with high scalability, compression rate, and fast decoding speed.

## Background

As integrity biological digital samples, vendor files are ideal for long-term storage for their high compression rate. However, due to cross-platform compatibility and software adaptation differences, Converting vendor files to other formats before data analysis is necessary. It is often discussed that converted files' accuracy is lossless or near-lossless. Data precision is mainly determined by the accuracy of the mass spectrometer and analytical parameters rather than the data's storage accuracy. Using too many digits for calculation leads to a waste of computing resources and lower calculation speed and software instability. Large files also bring a high cost of memory and bandwidth. In recent research, there are two directions on MS data compression. One is developing a new file format; the other is exploring a better data compressor.

The MS-Numpress [1] is a set of compressors providing algorithms with controllable precision. Considering the difference in the accuracy of intensity and m/z, MS-Numpress provides different near-lossless compression strategies. MassComp [2] presents a lossless compressor target on the m/z dimension, and it only works on mzXML [3]. The above two approaches are based on the mzXML or mzML [4] format, which cannot change the spectrum data index. Mz5 [5] and Toffee [6] are two new file formats. They both use HDF5 as their core storage technology. MzDB [7] uses SQLite database technology for storage, making the random access file reading more flexible and straightforward. However, without specific libraries or software, these files are not readable. A new format often brings much more additional work, such as adaptation to existing analysis software, building up software for file conversion, and compatibility with existing controlled vocabulary. However, when we use these data for calculation rather than long-term preservation, it is necessary to provide data with compatible precision and metadata information for software. Data not related to analysis is removed to reduce bandwidth and memory costs. Besides, the analysis software developers and the new format developers are often different. Therefore, the update progress of them is usually inconsistent. The software often fails to take advantage of the new format version's full capabilities.

We have developed Aird, a new file format that uses controllable precision and multiple index strategies to reconstruct the whole MS file, making it a more suitable data format for the computational process. The advantages of the Aird are as follows:

1. Faster decoding speed. Aird can decompress an m/z array of 400 MB per second [8];
2. Higher compression rate, which can significantly reduce memory usage and bandwidth;
3. More flexible file reading strategies by multiple indexing strategies, which can improve block read speed and reduce memory usage;
4. The metadata part stored as JSON format can be opened directly as a text format, which means users can preview the Aird file easily.

Aird is a double-file format that includes a metadata file and an MS data file. The metadata file contains the basic information from the vendor file and the fields from the controlled vocabulary stored as a JSON file. For the large MS data (mainly m/z-intensity pairs), Aird keeps it with controllable precision. Users can set the necessary accuracy when converting the vendor files. After determining the required precision, Aird converts the floating-point m/z array to an integer array. Because each spectrum has an ordered m/z collection, it is an effective way to compress relatively small deltas of adjacent integers instead of the large integers themselves. Aird uses the FastPfor library for differential coding of integer arrays [8]. The FastPfor library uses the SIMD technology, benefiting Aird to encode and decode the data faster with a high compression ratio.

We provide two tools for developers to use and understand Aird.

Aird SDK is the SDK for Aird data format accessing. Currently, we have developed the Java version of SDK.

AirdPro is a GUI client for data conversion from vendor file to Aird file. The vendor file reader API is from MSConvert [9].

## Methods

**Fig. 1:**
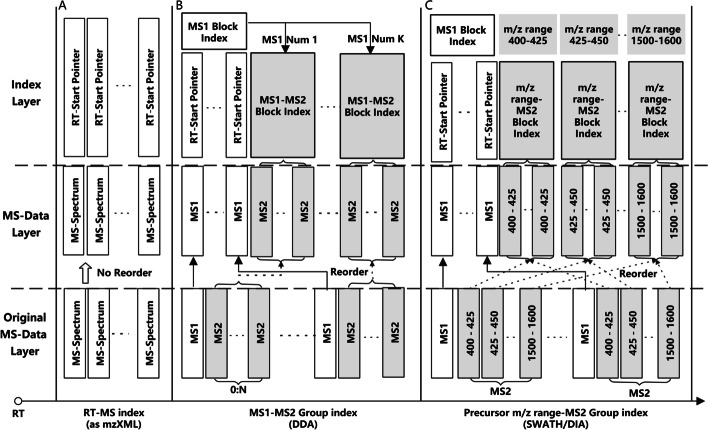
**A** Traditional mzXML-like indexing, MS data is sorted by retention time. The retention time and spectrum start position are stored as an index. **B** In DDA, each MS1 spectrum is followed by 0-N MS2 data. Extracted ion chromatogram is generally calculated in successive MS1 data, so reordering MS1 data to put them in the same physical file block can speed up file reads. **C** Typically used in SWATH/DIA, every MS2 data belongs to a particular m/z SWATH window. MS2 data in the same window will be frequently accessed and calculated. Reordering is essential for fast file I/O

An overview of the Aird data structure is shown in Fig. 1, and the main methods are described here:

### ZDPD algorithm for m/z compression

The m/z array has the following characteristics.

1. In metabolomics and proteomics, the fragment mass is generally between 0 and 3000;
2. The m/z arrays are ordered;
3. In the m/z array, there will be several consecutive numbers close to each other, and the difference of these numbers is relatively tiny and fixed;

We built a new compressor called ZDPD. The brief principle of ZDPD is shown in Fig. 3B. Aird chooses the integer type as the storage type for the m/z array. If the needed data precision is ten ppm (100 daltons to calculate), the accuracy can be accurate to 3 decimal places (dp). If 0.1 ppm is needed, five dp is necessary. The m/z array in each spectrum is an ordered array. Aird achieves the best compression rate by combining FastPfor and Zlib algorithms reasonably. FastPfor library is a suitable compressor to store deltas between adjacent integers, benefiting from its particular optimization. It also supports SIMD acceleration [8]. After compressing the sorted integers, Aird uses the Zlib algorithm(Compression Level = 6, Zlib library is DotNetZip from http://dotnetzip.codeplex.com) to compress the delta values, which can achieve a better result than only using the Zlib or FastPfor algorithm. The combination of the two algorithms perfectly utilizes the prior characteristics of the m/z array.

### Algorithm for intensity compression

Unlike the m/z array, the intensity data has a high degree of repeatability, consistent with the Zlib algorithm's compression characteristics. The results also show that Zlib has an excellent performance in compression of intensity array. Therefore, Aird chooses the Zlib algorithm as the intensity array's compressor. In AirdPro, we also implement another optional compression algorithm called "Log10 Compressor" proposed by MS-Numpress [1].

### Multiple indexing and storage strategies

Different acquisition modes have various computing features. For example, when dealing with DIA/SWATH. The most frequently calculated objects are the spectrums of the same precursor. They are discontinuous in retention time(RT). Using the traditional model based on RT will significantly reduce the reading efficiency and is unsuitable for distributed reading. According to these various features, Aird builds additional correlation indexes in the metadata so that the program can read the data more quickly during analysis.

Aird will take the calculation features of different acquisition modes as the core optimization point and provide a corresponding integration interface in SDK as a computing-oriented format. Aird also provides an RT-based data model that acts like mzML but has a much higher compression ratio.

Aird does not use any database technology like HDF5 or SQLite but also provides a similar querying ability. The spectrum querying ability strategies mainly depend on mass spectrometry acquisition modes. Different modes have different requirements for spectrum index. If we can summarize and adapt the indexes of varying acquisition modes, we can achieve the same query ability as using database technology.

The MS data storage includes the index strategy and the disk's actual storage location. For example, both mzXML and mzML provide an index strategy by storing the scanning number and start position of each spectrum, as it keeps specific MS data in the order of the scanning number. Each scanning number represents a particular RT. The indexing strategy of mzXML and mzML is inflexible. Considering the Data Independent Acquisition (DIA) and Data Dependent Acquisition (DDA), Aird described three indexing and storage strategies:

1. RT-MS index: This is one of the most traditional indexing strategies, which is also used in mzXML and mzML format. This strategy is an efficient way to access successive MS data. MS data are also linearly arranged by RT and stored in the file.
2. MS1-MS2 Group index: Considering DDA, each MS1 spectrum will correspond to zero or multiple MS2 data. Adjacent MS1 data are often taken out together to calculate the Extracted Ion Chromatogram (XIC). Therefore, Aird will reorder the spectrum data and put the data of MS1 together as one MS1 group. The MS2 groups corresponding to each MS1 will be put together. The MS1 group and MS2 group's start position will be added as the new index data.
3. Precursor m/z range-MS2 Group index: Considering DIA/SWATH, each MS2 spectrum corresponds to a specified precursor m/z range. Aird also builds specific index structures to improve block data's reading rate for a particular m/z range. The MS2 groups correspond to each specified precursor m/z range.

### The JSON format for metadata storage

JSON is a lightweight data exchange format with similar readability and extensibility as eXtensible markup language (XML). However, with the same semantics, JSON uses much less text than XML, making the JSON file smaller (See Fig. 3A). JSON is a subset of JavaScript, making it better in web performance and parsing speed. Like the mzXML Schema, we also provide a JSON Aird Schema (https://github.com/CSi-Studio/Aird-SDK/blob/1.1/AirdMetaData.json). A detailed description for every field in the Aird metadata is public on the Github page.

### Workflow for MS file converting

**Fig. 2:**
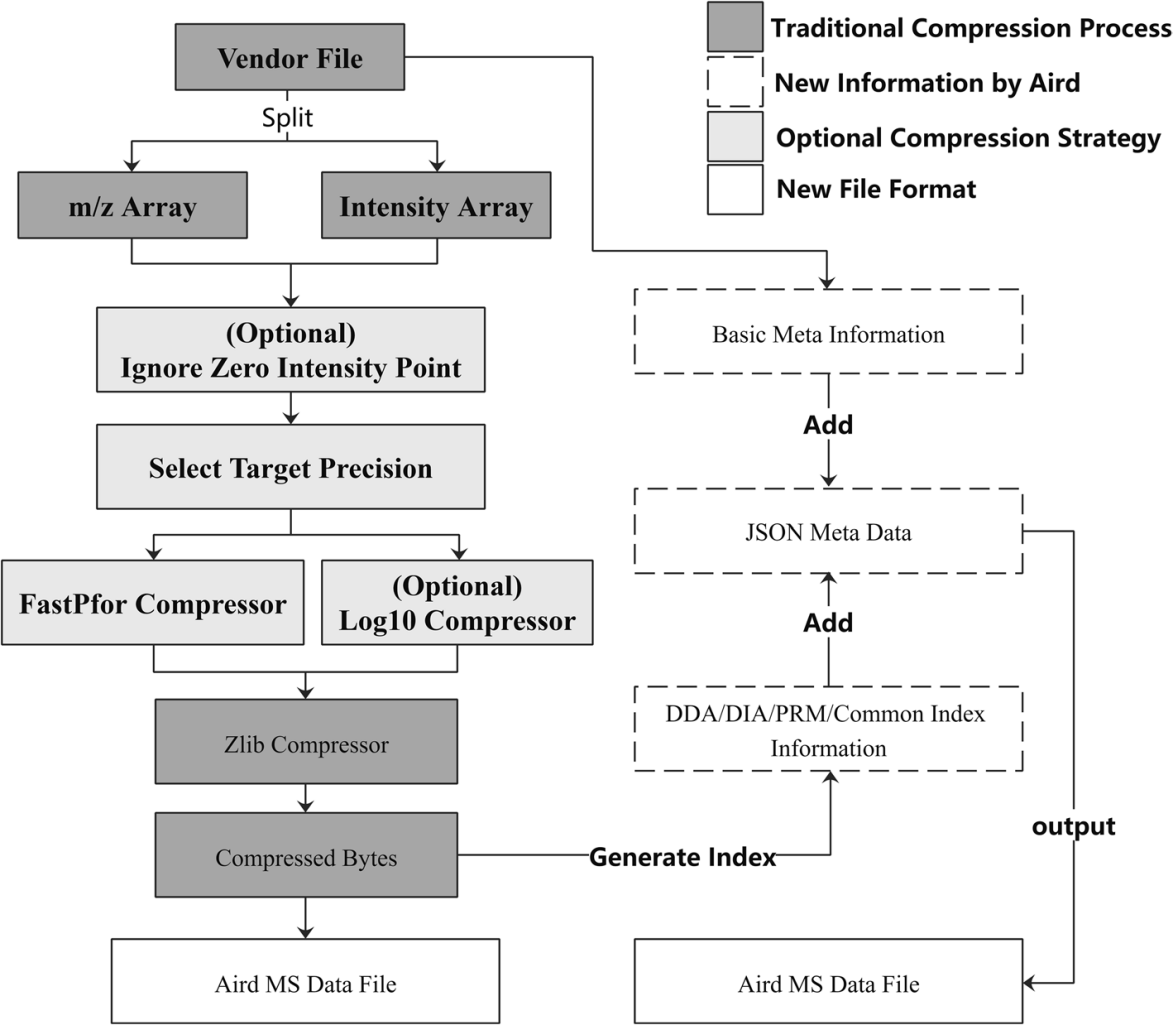
The conversion workflow for AirdPro. The picture shows how AirdPro separated the vendor files and compressed the m/z array and intensity array

AirdPro uses the integrated SDK provided by ProteoWizard [9] to read vendor file data for the first step of conversion. AirdPro starts to compress the m/z and intensity arrays according to the compression parameters and the user's targeted precision. According to the MS file's acquisition mode, AirdPro will reorder the spectrums for better random access performance. The detailed steps are shown in Fig. 2.

There are two optional steps in the workflow.

1. Ignore Zero Intensity Point. Aird would not store the zero-intensity pairs as most computation scenarios will not use these data. Researchers can unselect the option if you do need the zero-intensity data.
2. Log10 Compressor. The "log10 compressor" is an option method used to compress the intensity array. Suppose researchers do not require high accuracy of intensity. In that case, AirdPro provides a data processing method of log10 (intensity), which is a way to deal with the intensity array that has been put forward in MS-Numpress. We implement the same function in AirdPro to facilitate researchers in need.

## Results

We test the data on seven open datasets [10–16] with a normal desktop computer (CPU: i7 7700 K 4.4 GHz, Disk: 5900R HDD, Memory:16 GB). The MSConvert version is 3.0.20196-20896b6b1.

The parameters used in the mzML conversion:

Binary encoding precis: 32-bit;

Write index: Yes;

Use Zlib compression: Yes;

TPP compatibility: Yes;

Package in gzip: No;

Use numpress linear compression: 2.00E-09.

Use numpress short logged float compression: 0.0002.

The parameters used in the mz5 and mgf conversion:

Binary encoding precis: 32-bit.

Write index: Yes;

Use Zlib compression: Yes;

TPP compatibility: Yes;

Package in gzip: No;

The parameters used in the Aird conversion:

Is Zero Intensity Ignore: Yes.

Log2(Intensity): No;

m/z decimal places: 0.0001;

Since all the files are several terabytes in total, we selected a set of data files and uploaded them to the ProtemeXchange with PRIDE Submission Tool(PXD025142). The data files include the raw data and four converted files: mzML, mgf, mz5, and Aird file formats.

**Fig. 3:**
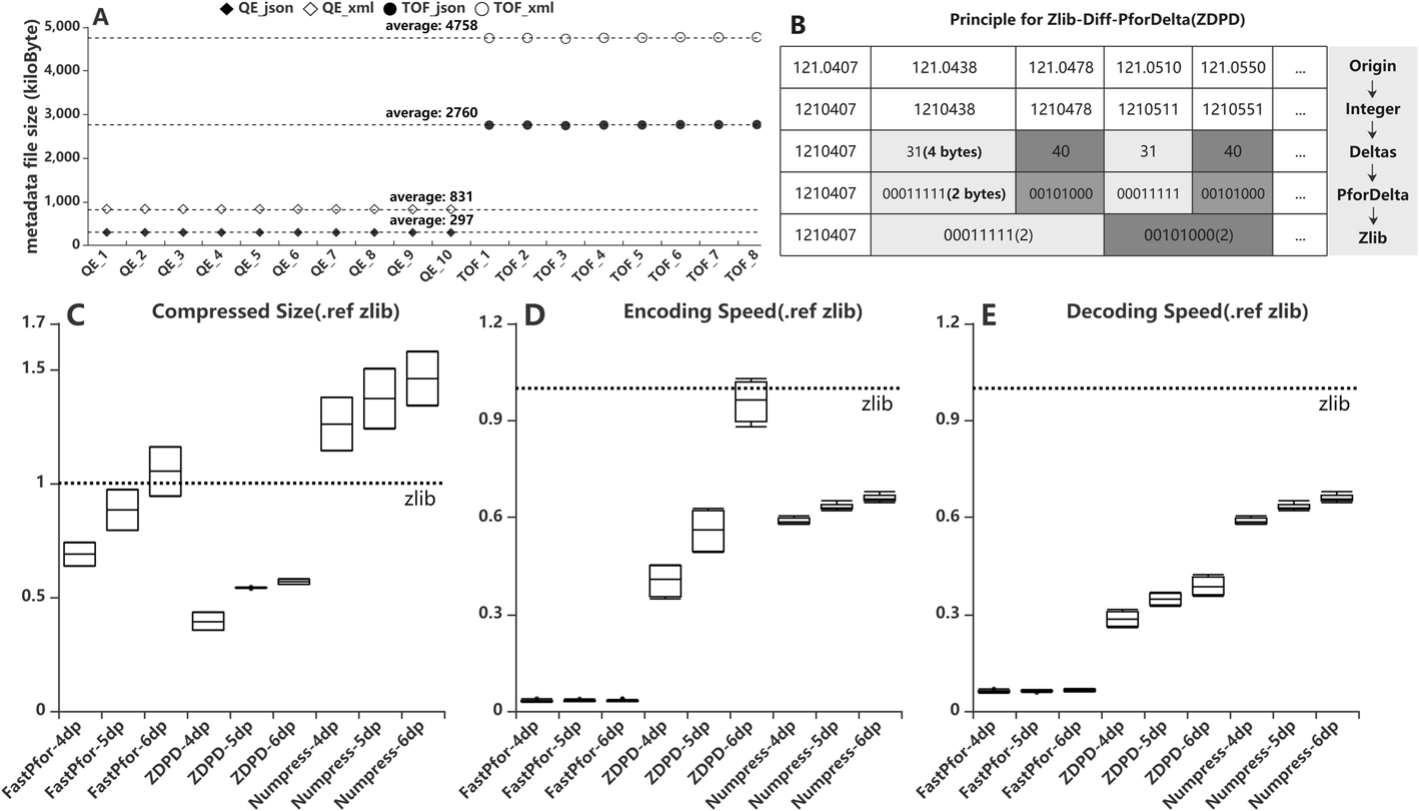
**A** Metadata with the same content is stored in JSON and XML format, and Thermo QE and SCIEX TOF generate the files. File size increased by about 65% when transferred from JSON to XML for SCIEX TOF files and 1.7 times for Thermo QE files. **B** The principle of the ZDPD algorithm. **C** The compression size is compared with Zlib using each compression algorithm. The ratio increases as the accuracy improve. The ZDPD algorithm shows the best performance. **D** The encoding time for each algorithm. Due to SIMD support, the FastPfor algorithm is high-speed. **E** The decoding time for each algorithm. Decoding time is one of the most critical parameters in the computation-oriented process. Although ZDPD is not the fastest, the ZDPD algorithm's performance is the most balanced when combining the compression rate and decoding speed rate

Metadata information is usually read into the memory for a quick preview of file information. Unlike MS data, metadata usually exists in memory for a long time, while MS data should be loaded into memory only during the calculation. However, metadata is generally less than 5 MB. But loading a project contains hundreds of experimental files. It will also take up more than 500 MB of memory. The JSON format can make file preview faster and reduce memory usage. XML and JSON formats are used to store the same content to compare the two formats' storage sizes (see Fig. 3A). The JSON format file is smaller than the XML format file due to eliminating redundant tag information in XML.

We compared m/z data size, encoding time, and decoding time with three different precisions. FastPfor, Aird, and Numpress are compared with Zlib (See Fig. 3C–E). In terms of actual size, the m/z data size compressed by the Aird algorithm is 55% of it of Zlib on average. When using OpenSWATH [17], MZMine [18], XCMS [19], or other software, XIC is a persistent and common calculation step. The increasing MS data size becomes more difficult to decode the MS data and load it into memory. Decoding speed has become one of the bottlenecks of the whole workflow. The ZDPD algorithm also performs well in decoding speed due to the SIMD support, which is only 33% of Zlib. That means, with the same I/O strategy, Aird uses only half of the memory to complete the calculation compared with Zlib but can increase the decompression speed by three times. For the intensity array, Aird uses the Zlib compressor with 1dp precision. As an option, Aird also offers an optional compression algorithm that can result in precision loss of up to 0.25%.

**Fig. 4:**
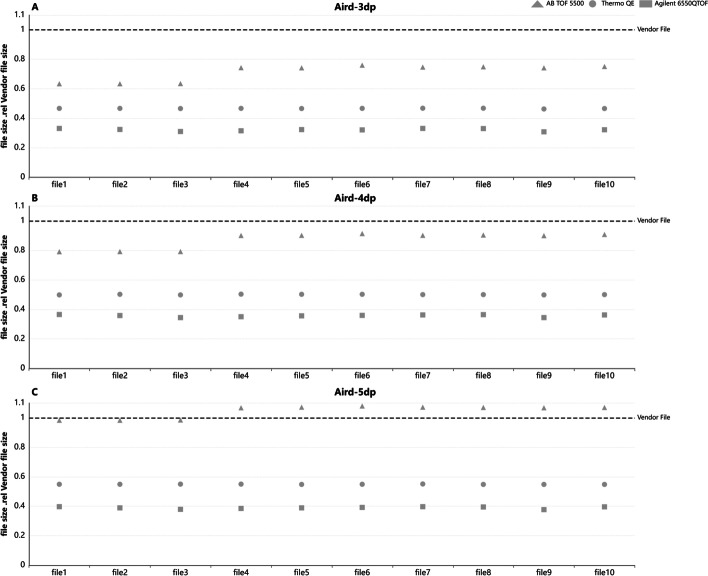
Aird's file sizes under different precision are compared with those of their manufacturers. We randomly select files from three manufacturers for comparison

**Fig. 5:**
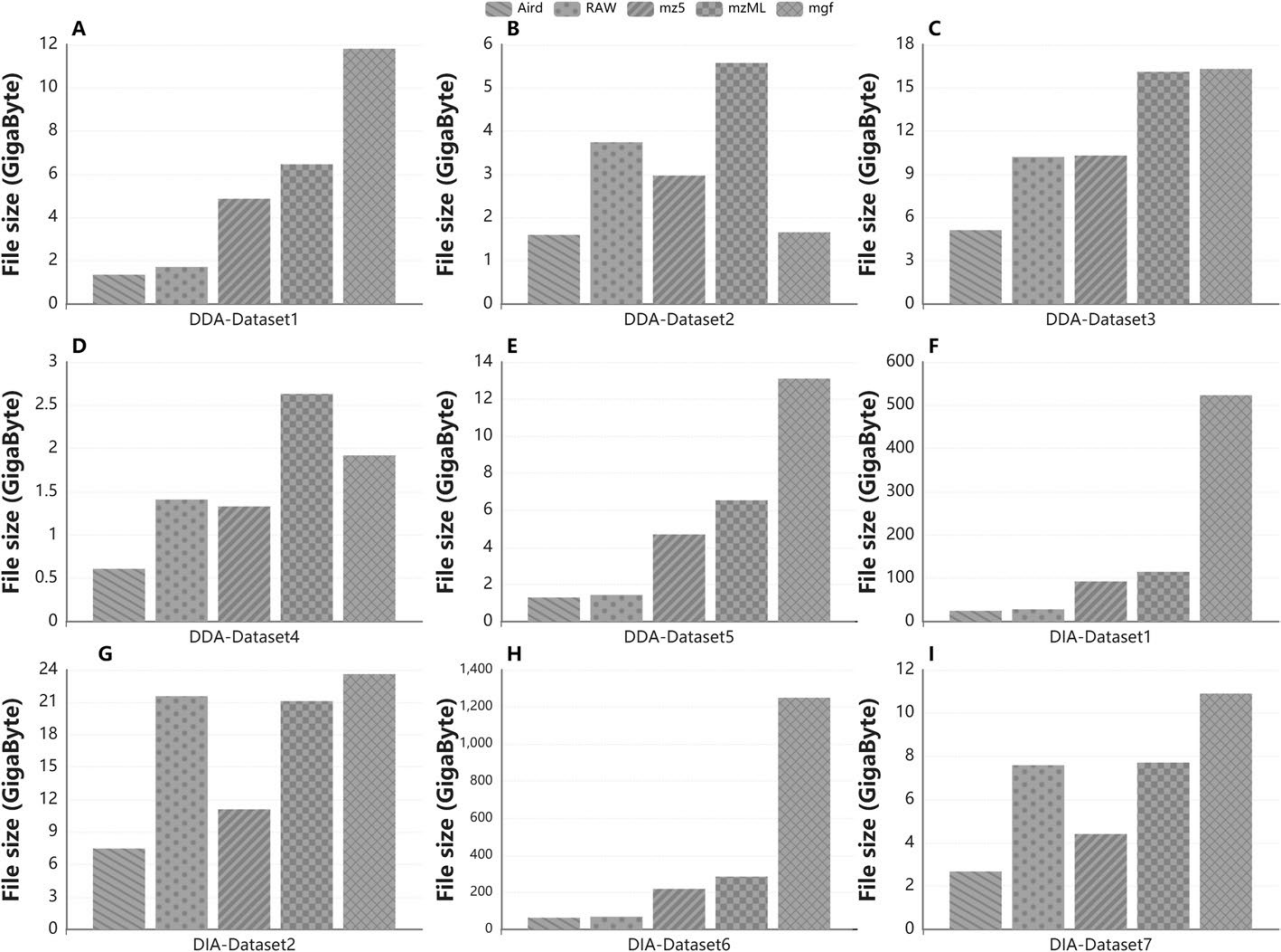
The comparison of file size between five formats in seven datasets. In these datasets. Dataset1 and Dataset2 contain both DDA and DIA files. The size of five datasets in DDA mode. In this mode, the average size of Aird files is 2.0 GB, which is 54% of RAW files. The size of four datasets in DDA mode. The average size of Aird files is 24.6 GB, 77% of RAW files. It means that Aird has the highest compression rate and takes the least storage space among the five formats

We compared the total file size on the open datasets. The compressed file size is divided by its vendor file for Aird of different precision for DDA files from SCIEX TOF 5500, Thermo QE, and Agilent 6550QTOF (see in Fig. 4A–C). File size increases as the accuracy improve. Compared with three dp Aird files, four dp and five dp of Thermo QE data increased by 7.3% and 17.7% on average. The increases for SCIEX TOF are 18.7% and 49.6%. The corresponding increases for Agilent QTOF are 10.9% and 21.5%. Also, Aird of 4dp is compared with mzML, vendor files, mz5, mgf for DIA and DDA files (see Fig. 5). It's worth noting that the file size of Aird is about 10%-20% less than the vendor file for data generated by SCIEX TOF, 50% when it comes to Thermo QE, and about 40% when compared with Agilent vendor format.

## Discussion

### Comparison ratio for compressor

Digital properties of intensity arrays vary across instruments. Duplicate intensities tend to be produced by the TOF spectrometer, which is a very favorable characteristic of the Zlib algorithm. Besides, m/z arrays with more numbers within the same range will have a higher compression rate as their delta values are minor. Therefore, under the same conditions, the more the number of fragments in the sample, the higher the compression rate. So we cannot come up with a stable and absolute conclusion to express Aird files' actual compression performance. It is also unfair to compare the total size between near-lossless and lossless formats. Although we provide some results regarding size comparison with mzML and vendor files, they are only for readers' reference. How to dynamically select the relevant compressor by using the acquisition modes generated by different types of the mass spectrometer is essential in the future.

### Plan for supporting scanning SWATH & ion mobility

At present, Aird's supporting formats are DDA, DIA/SWATH, PRM. ScanningSWATH and Ion Mobility are the formats under development. The support of each new acquisition mode requires the R&D team to deeply understand the data structure of the acquisition mode before building the corresponding index structure and the compression method for the newly added dimension data.

## Conclusions

In the omics calculation process for a multi-sample queue, part of the MS data will be read into memory frequently. However, it takes several terabytes of memory to load all files into memory at one time, which is too expensive. Solving the I/O and decompression performance bottleneck caused by frequent reading of MS data is challenging. On the other hand, the acquisition modes of MS are more abundant, and different mass spectrometers and different acquisition modes have additional requirements for accuracy. Using single or double precision to control the accuracy of mass spectrometry data in the calculation is insufficient. A new format with controllable precision for such a computing-oriented scenario is urgently needed. It should pay more attention to the decompression speed.

We have presented Aird, a computation-oriented MS data format consisting of two essential parts: the ZDPD compressor and the metadata storage model with JSON. The ZDPD compressor is for m/z values. We tested several datasets from different mass spectrometers and different acquisition modes, including proteomics data samples and metabolomics data samples. ZDPD can reduce the sizes by 49% than that by Zlib on average, and more importantly, ZDPD can decode the data 3 times faster than Zlib.

The existing MS data formats focus on whether the data is lossless and how high the compression rate is. In contrast, Aird pays more attention to exploring high decoding speed and controllable precision. The results show that ZDPD is a compressor with high performance for m/z, and for some controllable precision scenarios, Aird has firm compression and calculation performance. As a new MS data format member, Aird aims to speed up the MS data exchange and calculation ability and provide a unique solution for more computation-oriented MS data applications.

## Datasets description

See Table 1.

**Table 1:** The detailed description for each dataset

Datasets	Mode	Type	Instrument	Data ID
DDA-Dataset1	DDA	Proteomics	TripleTOF 5600	PXD021390
DIA-Dataset1	DIA	Proteomics	TripleTOF 5600	PXD021390
DDA-Dataset2	DDA	Proteomics	Q-Exactive	PXD018139
DIA-Dataset2	DIA	Proteomics	Q-Exactive	PXD018139
DDA-Dataset3	DDA	Proteomics	Q-Exactive	IPX0001509000
DDA-Dataset4	DDA	Metabolomics	Q-Exactive	MTBLS2119
DDA-Dataset5	DDA	Metabolomics	TripleTOF 6600	XCMS1197236
DIA-Dataset6	DIA	Proteomics	TripleTOF 5600 TripleTOF 6600	PXD002952
DIA-Dataset7	DIA	Proteomics	Agilent 6550	PXD004712

The PXD or IPX datasets can be searched on https://www.iprox.cn/, and the MTBL datasets can be found on https://www.ebi.ac.uk/metabolights/, the XCMS datasets can be searched on https://xcmsonline.scripps.edu/

AirdSDK is written in Java and is an open-source project for download at https://github.com/CSi-Studio/Aird-SDK. AirdPro is written in C# and is an opensource project for download at https://github.com/CSi-Studio/AirdPro/releases/.

## Data Availability

All data used in the manuscript is available online. The SWATH-MS Gold Standard Dataset [17] is from http://www.peptideatlas.org/PASS/PASS00289. Dataset1 is the peptide samples of the human 293 T lysate [12] from https://pubs.acs.org/doi/10.1021/acs.jproteome.0c00704. Dataset2 is the proteome samples of the coronatine [13] from https://www.frontiersin.org/articles/10.3389/fmicb.2020.01362/full. Dataset3 is the ginsenosides samples of cells [14] from https://pubs.acs.org/doi/10.1021/acs.jproteome.8b00972. Dataset4 is the yeast samples of cells [15] from https://www.sciencedirect.com/science/article/abs/pii/S0003267020310242. Dataset5 is the two standard mixtures SA and SB of drugs and metabolites dataset [10] from https://pubmed.ncbi.nlm.nih.gov/29907290. Dataset6 is the hybrid proteome samples of Human, Yeast, E.coli (HYE) [11] from https://www.ncbi.nlm.nih.gov/pubmed/27701404. Dataset7 is the FC2 samples of cells dataset during N-starvation [16] from https://pubmed.ncbi.nlm.nih.gov/28378827.

## Abbreviations

MS: Mass spectrometry
m/z: Mass to charge ratio
ZDPD: Zlib-Diff-PforDelta
SIMD: Single instruction multiple data
JSON: JavaScript Object Notation
RT: Retention time
DIA: Data independent acquisition
DDA: Data dependent acquisition
XIC: Extracted ion chromatogram
XML: Extensible markup language
dp: Decimal places

## Availability and requirements

This paper contains two software projects-AirdSDK and AirdPro. Project name: AirdSDK. Project home page: https://github.com/CSi-Studio/Aird-SDK. Operating system(s): Linux and Windows. Programming language: Java. Other requirements: Zlib-1.1.3 or above; FastPfor-0.1.12. License: Mulan Permissive Software License, Version 2. Any restrictions to use by non-academics: no. Project name: AirdPro. Project home page: https://github.com/CSi-Studio/AirdPro. Operating system: Windows. Programming language: C#. Other requirements:.NET Framework 4.7.2 or above;JSON-12.0.1; Zlib-1.1.3 or above;FastPfor 0.1.12. License: Mulan Permissive Software License, Version 2. Any restrictions to use by non-academics: no.
